# Intravenous Ketamine for Refractory Bronchospasm Precipitated by H1N1 Infection

**DOI:** 10.3389/fped.2014.00024

**Published:** 2014-04-02

**Authors:** Amit Agrawal, Jyotsna Shrivastava

**Affiliations:** ^1^Department of Pediatrics, Gandhi Medical College and Hamidia Hospital, Bhopal, India

**Keywords:** H1N1 infection, ketamine, wheezing, bronchospasm, acute severe asthma

## Abstract

Acute severe bronchospasm is an emergency situation and sometimes these children may fail to respond to conventional treatment and deteriorate rapidly to respiratory failure requiring mechanical ventilation. We present a case of 2-year-old girl, who presented with severe bronchospasm resulting in respiratory failure not responding to conventional management including mechanical ventilation and was found to be H1N1 positive. She was treated with ketamine infusion, which led to prompt improvement in airway obstruction.

## Introduction

Wheezing in children is due to narrowing of the intrathoracic airways (bronchospasm) from illnesses like asthma and bronchiolitis, resulting in limitation of expiratory air flow. A recent study showed that wheezing was present in up to 43.8% of all pandemic influenza-A (H1N1) cases and was associated with influenza mortality (1). Acute exacerbation of asthma in H1N1 infected cases is usually diagnosed in presence of wheezing and history of asthma. Previous studies have shown that asthma was the most common condition present in both children as well as adults hospitalized due to H1N1 viral infection, which worsens the respiratory symptoms in these cases (2, 3). On the other hand, H1N1 infection is found to provoke severe exacerbations in asthmatic and atopic children (3, 4).

Children with acute exacerbations sometimes fail to respond to standard therapy (i.e., inhaled beta2-agonist and anticholinergics and oral or parenteral corticosteroids) or adjunctive therapies such as intravenous magnesium, methylxanthines, beta-agonists, heliox, and non-invasive ventilation requiring invasive ventilation with associated morbidities (e.g., barotrauma and prolonged hospitalization) (5). Ketamine has been shown to be effective in these refractory cases both in adults and children (6). Ketamine is a powerful bronchial relaxant, which relieves bronchospasm and improves pulmonary compliance in patients with reactive airway disease and bronchospasm by increasing catecholamine release and inhibiting catecholamine reuptake processes. We, hereby, report successful use of ketamine in a child with acute asthma exacerbation secondary to H1N1infection, who failed to respond to standard treatment.

## Case Discussion

A 2-year-old girl was admitted with complaints of cough and high grade fever for 4 days and breathing difficulty for 2 days. She had history of repeated episodes of respiratory illnesses for which she received nebulization therapy and responded well. However, no documentation of medications was available and she was not on regular inhaled or oral medication. There was no family h/o atopy or asthma. On admission, she was irritable, febrile (102.6°F), tachypneic (respiratory rate 56/min) with subcostal and intercostal retractions and heart rate was 130/min with normal perfusion. Her oxygen saturation (SpO2) on room air was low (86%). Respiratory system examination revealed bilaterally equal and adequate air entry, bilateral wheezing, and occasional crepitations. Other systemic examination was unremarkable except for irritability.

Based on history and examination, initial diagnosis of acute exacerbation of asthma was made and treatment was started with humidified oxygen and salbutamol nebulization along with other supportive measures. IV antibiotics (third generation cephalosporin and aminoglycosides) were started empirically in view of high grade fever. On complete hemogram, hemoglobin was 10 g/dl, total leukocyte count – 16000/mm^3^, and differential count (P – 54%, L – 35%, M – 6%, E – 4%, B – 1%). However, bacteriological culture was negative and other basic laboratory investigations were within normal limits. Chest X-ray revealed bilateral hyperinflation.

However, despite these measures, she continued to deteriorate with increasing respiratory distress and SpO2 was varying between 86 and 92%. Therefore, she was shifted to pediatric intensive care unit (PICU) for further management and was treated sequentially with ipratropium and budesonide nebulization, IV hydrocortisone and magnesium sulfate, subcutaneous adrenaline, and IV infusion of terbutaline and aminophylline over next 10 h. However, her condition continued to worsen with no improvement in respiratory distress. Her arterial blood gas analysis revealed hypoxemia and respiratory acidosis; therefore, she was intubated and put on mechanical ventilation.

Despite several ventilatory changes and supportive measures over next 12 h, her bronchospasm persisted, SpO2 and ABG parameters were worsening. Therefore, we decided to use IV ketamine as a last measure after taking consent from the parents. Ketamine was given as initial bolus (0.5 mg/kg) followed by continuous infusion (0.5 mg/kg/h), which increased gradually up to 2 mg/kg/h. The use of ketamine was not preceded by atropine. Within 4 h, she started showing response in the form of decrease in wheezing and improvement in peak inspiratory pressure, gas exchange, and SpO2. The aminophylline, terbutaline, and magnesium infusions were tapered off gradually in 24 h. Her throat swab sent for H1N1 turned out to be positive so oseltamivir therapy was initiated; however, repeat chest X-ray showed only hyperinflation with few infiltrates. She showed gradual improvement in clinical condition and 72 h later, she was extubated to non-invasive ventilation for next 24 h. Ketamine infusion was continued for 72 h and then gradually weaned with no side effects of therapy. She was shifted out of PICU after 5 days and was discharged to home after another 2 days.

## Discussion

In the present case, we successfully used ketamine infusion for refractory bronchospasm in a child with acute asthma exacerbation precipitated by H1N1 infection. Previous studies have suggested that asthma may be a risk factor that worsens respiratory symptoms during pandemic H1N1 infection and occurrence of asthma exacerbation might have been increased by H1N1 infection (1–4). The index case also had history suggestive of asthma which most probably was precipitated by H1N1 infection. Previous study showed that children with pandemic H1N1 infection were more likely to have asthma than children with seasonal influenza (22 vs. 6%) and usually they present with wheezing and bronchospasm (4).

Owing to its bronchodilatory effect, ketamine has been successfully used to treat patients with severe bronchospasm unresponsive to conventional (e.g., beta2-agonist, anticholinergics, and corticosteroids) as well as adjunctive therapies (e.g., IV magnesium, methylxanthines, and beta-agonists). Many studies demonstrated the beneficial use of ketamine for acute exacerbation of asthma in pediatric patients, both non-ventilated and ventilated (6–8). A recent review showed a favorable response to ketamine in most of the studies (5). In non-intubated patients with severe respiratory distress, ketamine obviated the need for mechanical ventilation with decrease in wheezing and improvement in SpO2 and blood gas. Mechanically ventilated patients showed improved peak inspiratory pressures, dynamic compliance of lungs, gas exchange, and decreased oxygen requirements (6–8).

The suggested mechanisms of airway relaxation include ketamine induced inhibition of catecholamine reuptake, increased catecholamine concentrations, voltage-sensitive Ca^++^-channel blockage, and inhibition of postsynaptic nicotinic or muscarinic receptors. Various authors used ketamine as bolus doses ranging from 0.1 to 2 mg/kg followed by continuous infusion (0.15–2.5 mg/kg/h) with no major adverse effects (5, 6). Minor adverse effects reported include dysphoria, hallucinations, increased secretions, and mild perturbations in heart rate and blood pressure in few patients.

To conclude, present case further supports the use of intravenous ketamine as an effective temporizing measure for children experiencing severe asthma exacerbations refractory to conventional therapy. However, its place in status asthmaticus cannot be overemphasized where most of the patients respond to conventional drugs and large scale randomized controlled trials are needed to establish its role as a bronchodilator.

## Conflict of Interest Statement

The authors declare that the research was conducted in the absence of any commercial or financial relationships that could be construed as a potential conflict of interest.
